# Increased cross-modal functional connectivity in cochlear implant users

**DOI:** 10.1038/s41598-017-10792-2

**Published:** 2017-08-30

**Authors:** Ling-Chia Chen, Sebastian Puschmann, Stefan Debener

**Affiliations:** 1 Neuropsychology Lab, Department of Psychology, European Medical School, University of Oldenburg, Oldenburg, Germany; 2 Cluster of Excellence Hearing4all, Oldenburg, Germany; 3 Research Center Neurosensory Science, University of Oldenburg, Oldenburg, Germany; 4 Biological Psychology Lab, Department of Psychology, European medical school, University of Oldenburg, Oldenburg, Germany

## Abstract

Previous studies have reported increased cross-modal auditory and visual cortical activation in cochlear implant (CI) users, suggesting cross-modal reorganization of both visual and auditory cortices in CI users as a consequence of sensory deprivation and restoration. How these processes affect the functional connectivity of the auditory and visual system in CI users is however unknown. We here investigated task-induced intra-modal functional connectivity between hemispheres for both visual and auditory cortices and cross-modal functional connectivity between visual and auditory cortices using functional near infrared spectroscopy in post-lingually deaf CI users and age-matched normal hearing controls. Compared to controls, CI users exhibited decreased intra-modal functional connectivity between hemispheres and increased cross-modal functional connectivity between visual and left auditory cortices for both visual and auditory stimulus processing. Importantly, the difference between cross-modal functional connectivity for visual and for auditory stimuli correlated with speech recognition outcome in CI users. Higher cross-modal connectivity for auditory than for visual stimuli was associated with better speech recognition abilities, pointing to a new pattern of functional reorganization that is related to successful hearing restoration with a CI.

## Introduction

Cortical changes induced by sensory deprivation have been robustly reported in blind and deaf individuals^1–3^. Specifically, the lack of visual or auditory sensory input induces a shrinkage of the corresponding cortical representations, and is usually paralleled by an invasion of the spared sensory modalities^4–7^. In the case of deafness, modern cochlear implant (CI) technology allows a partial hearing recovery^8^. Hearing recovery with CIs also induces cortical reorganization that may be qualitatively different from changes previously induced during the period of deafness. Specifically, CI users have shown higher cross-modal cortical activation in both visual and auditory regions when compared to normal hearing (NH) controls^9–12^. It is often suggested that this pattern of reorganization of auditory cortex reflects leftover cortical changes previously induced by deafness. On the other hand, a recruitment of visual cortex for auditory processing presumably reflects cortical changes associated with the adaptation to the new CI input. This distinction is important, as strong cross-modal reorganization of auditory cortex impacts negatively on CI outcome^9, 10^, whereas cross-modal changes in visual cortex seem to support speech understanding in CI users^9, 12^.

The mechanisms underlying cross-modal reorganization in CI users remain poorly understood. One theory suggests that the process is mediated by the enhancement of pre-existing connections between visual and auditory cortex^2, 13, 14^. Supporting evidence comes from animal studies showing increased anatomical projections from visual to auditory cortices in both early- and late-deaf versus normal-hearing cats^15–17^, even though these changes are very specific and the general pattern of cross-modal connectivity seems not significantly altered in deaf animals^16, 18^. In humans, it has also been found that visual input can modulate auditory responses, which is compatible with the idea of direct cross-modal interactions^19, 20^. Alternatively, such mediation could be implemented through a separate multi-sensory region, such as in parietal cortex. Indeed enhanced structural connectivity between parietal cortex and low level visual cortices has been observed in deaf individuals^21, 22^. Importantly, both theories predict increased functional connectivity between visual and auditory areas in both deaf individuals and CI users. Unfortunately, in sharp contrast to numerous structural and task-induced functional connectivity studies in blind and deaf individuals^23–27^, little is known about cross-modal connectivity in CI users, despite the significance of such observations in suggesting an increase of information flow between visual and auditory cortices. Reasons for this lack of evidence are mostly related to safety concerns with regards to metal implants in a magnetic resonance imaging scanner, which precludes a proper assessment of structural or functional connectivity in CI users.

Functional near-infrared spectroscopy (fNIRS) provides the unique opportunity to close this gap. A recent fNIRS study reported for the first time decreased resting state functional connectivity between left and right auditory areas in CI users compared to NH controls^28^. This work demonstrates that fNIRS can be used to assess potential changes in functional connectivity patterns in CI users. In the current study we therefore re-analyzed a previous fNIRS dataset in which CI users and controls were presented with visual and auditory stimuli^9^. The aim was to investigate whether task-induced cross-modal functional connectivity between visual and auditory cortices is indeed elevated in CI users as predicted from the possible mechanisms underlying cross-modal reorganization. Additionally, following the recent resting state connectivity results^28^ we expected decreased task-induced intra-modal functional connectivity between hemispheres in both visual and auditory areas in CI users compared to NH controls, reflecting intra-modal plasticity in CI users. Since cross-modal reorganization may help to explain variability in clinical outcome^9, 10^, we assessed the relationship between cross-modal functional connectivity and speech recognition abilities in CI users. Specifically, with previous evidence pointing towards a negative role of cross-modal activation in auditory cortex and a positive role of cross-modal activation in visual cortex, we predicted speech recognition to have a positive relationship with cross-modal connectivity for auditory stimuli and a negative relationship with cross-modal connectivity for visual stimuli.

## Materials and Methods

### Participants

Forty adults (fourteen males, twenty-six females) comprising twenty post-lingually deaf CI users and twenty NH individuals participated in the study. All participants had normal or corrected-to-normal vision, and none had a history of neurological or psychiatric illness. Four participants were left-handed and the others were right-handed as assessed by the Edinburgh Handedness Inventory^29^. One CI user was excluded due to contamination of noisy signal from movement. The remaining nineteen CI users were all unilaterally implanted, three in the left ear and the other sixteen in the right. All CI users had been continuously using their devices for at least 6 months prior to the experiment (Mean 5.03 ± 3.75 years, range 0.5 to 16 years, Table 1). Because of the considerable age variance across all CI users (Mean 54.58 ± 14.96, range 24 to 77 years), each CI user was matched with a NH individual for gender, age (±3 years), handedness and education level. The NH participants (Mean 54.89 ± 15.80, range 24 to 78 years) served as controls and were tested for hearing abilities. One NH participant (not a matched control for the excluded CI user) was excluded due to extensive movement during the experiment. All participants provided written informed consent prior to the experiment. All procedures were approved by the local ethics committee of the University of Oldenburg and conformed to the declaration of Helsinki.

**Table 1:** Subject characteristics for cochlear implant users.

Subject	Gender	Age	Implant ear	Deafness duration (years)	CI usage (years)	OLSA_q (%)	OLSA_n (dB)
1	M	24	R	14	7	99,30	−2,3
2	M	47	R	0.25	3	21,30	N/A
3	F	51	R	2	7	100,00	0,9
4	F	22	R	8	2	98,00	−2,6
5	M	63	R	2	1	94,00	1,9
6	F	67	R	6	16	96,70	−1,9
7	F	49	L	25	6	96,00	−2,1
8	M	71	R	27	5	80,70	1,7
9	F	58	R	1	10	89,30	0
10	F	57	R	10	5	98,00	−0,4
11	F	59	R	4	2	91,30	0,1
12	F	77	R	13	6	100,00	−1,1
13	M	64	R	3	3	76,00	3,3
14	F	52	L	8	5	94,70	−2,6
15	F	36	R	1	9	93,30	0,2
16	M	70	R	7	2	93,30	−0,4
17	F	58	R	2	2	82,70	1,1
18	F	46	L	5	4	100,00	−1,8
19	F	66	R	0.5	0.5	78,70	3,1

### Experimental design

The visual stimuli were circular checkerboard patterns adopted from a previous study^10^. Checkerboards (image dimensions are 1024 × 1024 pixels) were radial and consisted of twenty rings, each of which was divided into 18 sectors of interchanging white and black color. This layout compensated for the increase in receptive-field size with eccentricity^30, 31^. There were four pairs of checkerboard patterns with different proportions of white pixels, corresponding to luminance ratios of 12.5%, 25%, 37.5%, and 50%. The contrast between white and black pixels was identical in all images. Contrast reversals were presented at 2 Hz for 10 seconds. All visual stimuli were presented on a 24-inch monitor at a distance of 150 cm. The visual angle of the checkerboards was 10.5°.

Auditory stimuli consisted of words, reversed words and tones were all sampled at 44.1 kHz. Disyllabic German words each had a duration of 800 ms and were adopted from a previous study^32^. The reversed words were the same German words played backwards. The goal here was to compromise the intelligibility but maintain the overall spectral properties. Word trains of three consecutive words/reversed words were presented with an inter-stimulus interval of 1.3 s and a total duration of 5 s. Within each word train, the words/reversed words were either repeated or not repeated, resulting in four conditions (words/reversed words x repeated/unrepeated) in total. Tones, adopted from Zhang and colleagues (2011), were 1 kHz pure tones of 40 ms duration with 5 ms linear onset and offset ramps. Bursts of five tones were presented with an inter-stimulus interval of 0.7 s and a total duration of 3 s. All auditory stimuli were presented through two loudspeakers located in front of the participants, 21 degrees to the right and to the left at ear height. The loudness level for both tones and words was adjusted individually to a subjectively-reported comfortable loudness level.

**Figure 1:**
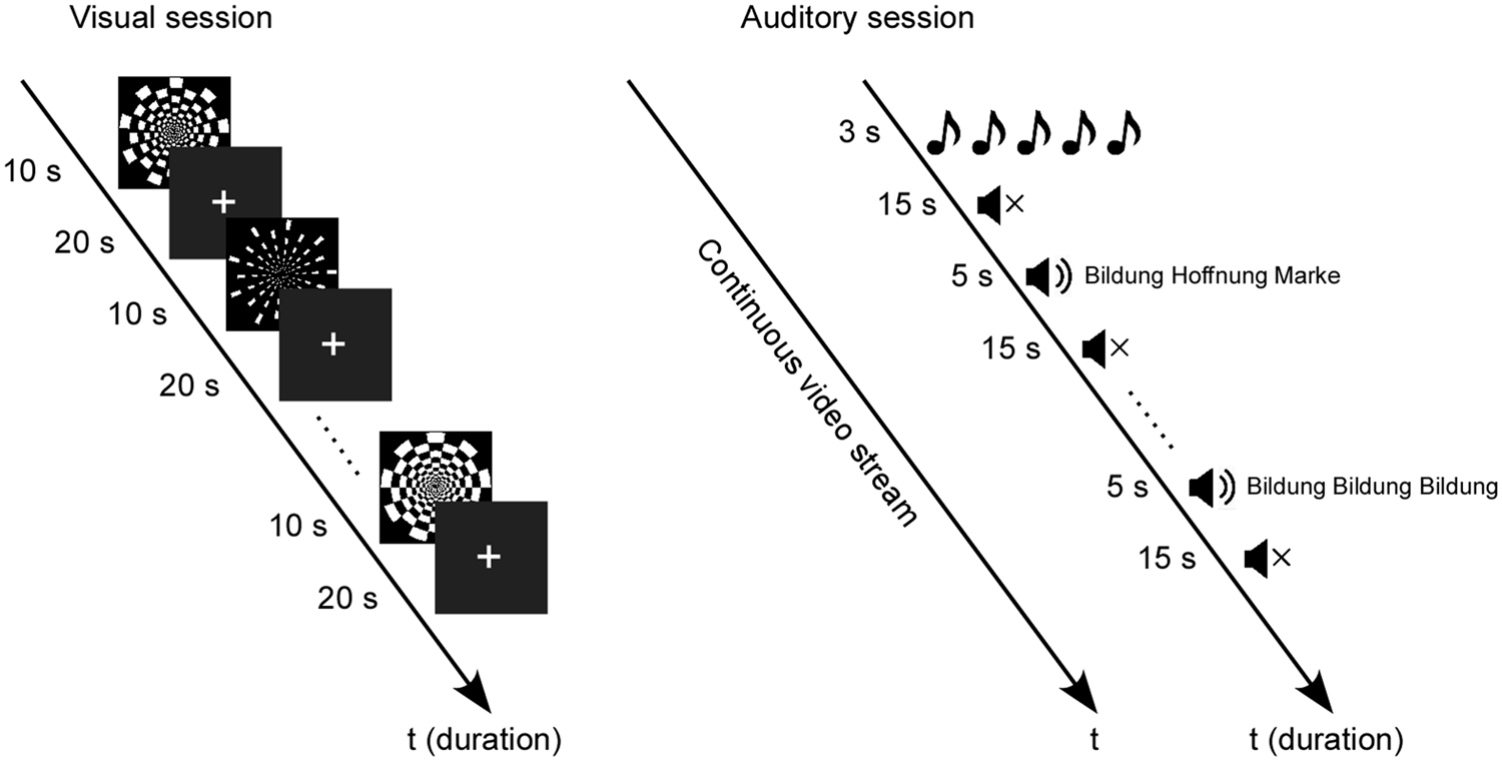
Paradigm. The visual paradigm is depicted on the left and the auditory paradigm is depicted on the right. In the visual session, flickering checkerboards were presented for 10 s followed by a 20 s baseline period with a fixation cross. In the auditory session, a continuous video is shown in the middle of the screen, while auditory stimuli were presented in the background. Each type of auditory stimuli (tones, words, reversed words) was followed by 15 s silence period and the sequence of presentation was random.

The experiment consisted of a visual and an auditory session (Fig. 1). In the visual session, forty blocks (four luminance ratios × ten repetitions) were presented. Each block consisted of one luminance ratio pair flickering at 2 Hz for 10 s, followed by a 20 s baseline period with a fixation cross in the center of the screen. The visual session lasted for around 20 mins, and a break of 1 min was given after the first 10 mins. In the auditory session, thirty blocks of tone bursts and sixty blocks of word trains (four conditions × fifteen repetitions) were presented. The tone blocks consisted of 3 s stimuli followed by 15 s of silence and the word train blocks consisted of 5 s stimuli followed by 15 s of silence. Due to the long periods of non-stimulation periods necessary for block design, a passive listening task was implemented to control for attention level^33–35^. A silent nature documentary was presented in the center of the screen during the auditory session, which lasted for around 30 mins. Within both visual and auditory sessions, all the blocks were randomly presented in sequence. The order of the visual and the auditory sessions was counterbalanced across participants.

### Procedure

All participants were screened for normal or corrected-to-normal vision, defined as a visual acuity of at least 0.6 with a Landolt C vision test. The NH controls passed a hearing threshold test indicating less than 30 dB hearing loss in each ear (125–4000 Hz). All participants answered a set of questionnaires including handedness^29^ and health state. CI participants additionally answered a questionnaire of CI-related questions such as the duration of deafness or CI implantation. For the visual session, participants were instructed to fixate at the center of the screen continuously, and to press a button at the end of the stimulus to indicate whether the stimulus belonged to a higher (50% or 37.5%) or to a lower (25% or 12.5%) luminance ratio. In the auditory session, participants were instructed to fixate at the center of the screen and to avoid saccades as much as possible (closing eyes was discouraged). The task was to focus on the silent documentary and to ignore the sound presentation. Participants were informed that after the experiment a questionnaire would be given related to the video content and that the answers would be evaluated. Prior to the data recording, the participants received training for the visual task, which was repeated until the hit rate exceeded 75%. After the experiment, CI users performed three speech recognition tests which were the Freiburg monosyllabic words test^36^, the Oldenburg sentences test (OLSA) in quiet, and the OLSA test in noise^37^. All speech recognition tests were auditory only without any visual component. The Freiburg monosyllabic word test measures single word recognition and consists of twenty lists of monosyllabic words. One list was randomly selected for each measurement, and the percentage of correctly repeated words by the participant was calculated as the test score. The OLSA test measures word recognition within a sentence structure. The OLSA test in quiet (OLSA_q) measures the percentage of correctly identified words within a sentence at a sound intensity level of 65 dB SPL. The OLSA test in noise (OLSA_n) uses an adaptive procedure to estimate the speech recognition rate, which was defined as the signal-to-noise ratio at which the participants achieved 50% correctly identified words within a sentence. The OLSA_n test was only performed for participants who achieved at least at 50% correct rate in the OLSA_q test to avoid frustration for the participants, since speech recognition in noise is dramatically more difficult and demanding compared to speech recognition in quiet. This resulted in one CI user without an OLSA_n test score (Table 1). NH controls also performed the OLSA_n test.

### Data recording

**Figure 2:**
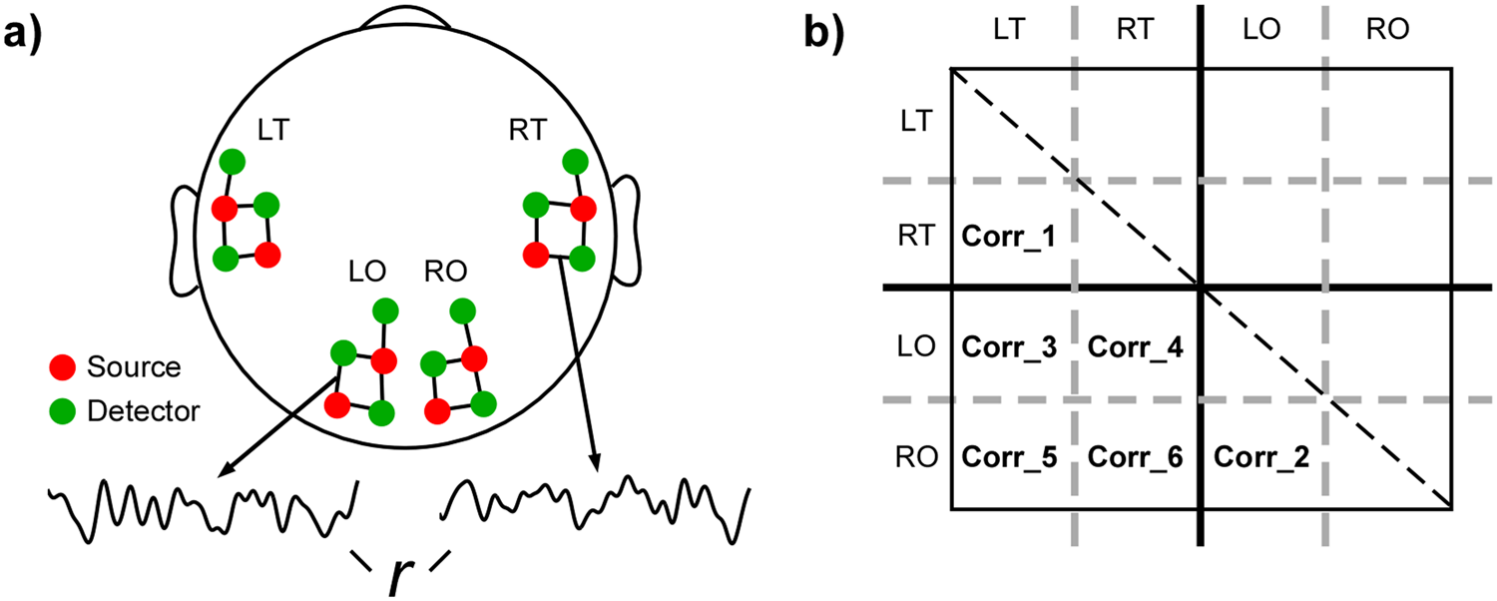
Calculation of functional connectivity in fNIRS. (**a**) Optode layout. The red and green circles indicate sources and detectors respectively. Optodes were placed over the left (LT) and right temporal lobes (RT) for measuring auditory responses and over the left (LO) and right occipital lobes (RO) for measuring visual responses. Each line connecting neighboring source and detector defined a measurement channel, resulting in 5 channels per region. The time course of each channel was correlated with all other channels and the resulting r values formed the correlation matrix shown in (**b**). (**b**) Correlation matrix and definition of regions of interest (ROI). The correlation matrix is a symmetrical matrix with ones on the diagonal (dashed line) and mirrored values above and below the diagonal. The matrix indices are in the sequence of 5 left temporal (LT) channels, 5 right temporal (RT) channels, 5 left occipital (LO) channels, and 5 right occipital (RO) channels. As a result, Corr_1 and Corr_2 in the upper left and lower right quadrant both indicate intra-modal connectivity, whereas Corr_3, Corr_4, Corr_5, Corr_6 in the lower left quadrant reflect cross-modal connectivity.

Functional near-infrared spectroscopy (fNIRS) was recorded using a NIRScout 816 device (NIRxMedizintechnik GmbH, Berlin, Germany) with eight LED sources (intensity 5 mW/wavelength) and twelve detectors. The optodes were placed over the left and the right temporal areas, centered around positions T7 and T8 in the 10–20 system, and over the left and the right occipital areas, centered around O1 and O2. Within each area, two sources and three detectors were placed, with each source and its neighboring detector being separated by 3 cm (Fig. 2a). Each source-detector pair formed a channel, resulting in five channels per area and twenty channels in total (Fig. 2a). The emitted light wavelengths were 760 nm and 850 nm, and the light intensity acquired at the detectors was sampled at 6.25 Hz. Electroencephalography (EEG) data were simultaneously recorded with fNIRS and the results reported elsewhere^38^.

### Data processing and statistical analysis

The fNIRS data were imported into Matlab and transformed to concentration levels (unit: mM) of oxygenated (HbO) and deoxygenated hemoglobin (HbR) using the NILAB toolbox (NIRxMedizintechnik GmbH, Berlin, Germany). Since HbR concentrations are thought to be more spatially specific than HbO concentrations^39–41^, only HbR concentrations were analyzed in the current study. In four CI users, two channels were blocked by the transmitter of the CI device and had to be excluded from subsequent analyses. In addition, all channels selected in the previous report of this same dataset (Chen *et al*., 2016) were excluded. In this previous study only channels with the highest beta values estimated from a general linear model (GLM) in occipital and temporal areas for visual and auditory stimuli were statistically analyzed. This was done for two reasons. Firstly, most previous animal studies suggest that cross-modal take-over in deafness may be strongest in the secondary visual and auditory areas rather than primary areas^7, 42^. We reasoned that channels showing highest activation for visual checkerboard and auditory tone stimuli could be functionally identified as capturing responses from more primary visual and auditory cortices. Thus by excluding these channels our analysis focused better on secondary visual and auditory areas. Secondly, since this same data set has been previously reported showing higher cross-modal activation in both visual and auditory areas in CI users compared to NH controls^9^, this allowed us to explore the unreported data.

In contrast to the previous report focusing on condition effects, here the stimulations were treated as continuous input streams to study general connectivity patterns. That is, the entire time series from the remaining channels, which were not included in the previous publication stemming from the same dataset^9^, were correlated to all other channels using Pearson correlations. This was done separately for the 20 minutes visual session time series and for the 30 minutes auditory session time series. A Fisher’s z transformation was used to transform the resulting r values to z values for subsequent analysis. Fisher’s z values were averaged separately to estimate intra-modal connectivity and cross-modal connectivity. Specifically, intra-modal connectivity consisted of correlations between left and right auditory areas (Corr_1) and between left and right visual areas (Corr_2), and cross-modal connectivity consisted of correlations between left visual and left auditory areas (Corr_3), left visual and right auditory areas (Corr_4), right visual and left auditory areas (Corr_5), and right visual and right auditory areas (Corr_6). The z values were averaged separately for visual and auditory stimuli conditions (Fig. 2b).

Repeated measures three-way ANOVAs, with factors stimulus type (auditory, visual) and modality (cross-modal, intra-modal) as within-subject factors and group (CI, NH) as a between-subject factor, were performed to investigate differences in cross-modal and intra-modal functional connectivity between CI users and NH controls. Since previous studies showed evidence of hemisphere-specific cross-modal reorganization of auditory cortex in post-lingually deaf CI users^6, 10, 43^, two separate ANOVAs were performed. In one ANOVA Fisher’s z values were averaged across Corr_3 and Corr_5, representing cross-modal connectivity between left auditory area and both left and right visual area. Similarly, in the other ANOVA Fisher’s z values were averaged across Corr_4 and Corr_6, that is, the cross-modal connectivity between the right auditory area and both left and right visual area. For both ANOVAs, the intra-modal connectivity for visual stimuli was obtained by averaging Fisher’s z values across Corr_2, representing connectivity within visual areas between hemispheres, and for auditory stimuli by averaging Fisher’s z values across Corr_1, representing connectivity within auditory areas between hemispheres (Fig. 2b). All statistic tests were two-tailed and the alpha-level was set at 0.05.

To investigate the relationship between cross-modal connectivity and speech recognition, cross-modal connectivity (Corr_3–6) was first normalized by subtracting intra-modal connectivity (Corr_1 & 2) to account for individual differences. Additionally, since cross-modal connectivity for visual and for auditory stimuli was predicted to be negatively and positively associated with speech recognition performance respectively, the normalized cross-modal connectivity for auditory stimuli was subtracted from cross-modal connectivity for visual stimuli and correlated with the individual speech recognition ability using Spearman’s Rho correlation to account for non-linearity.

## Results

**Figure 3:**
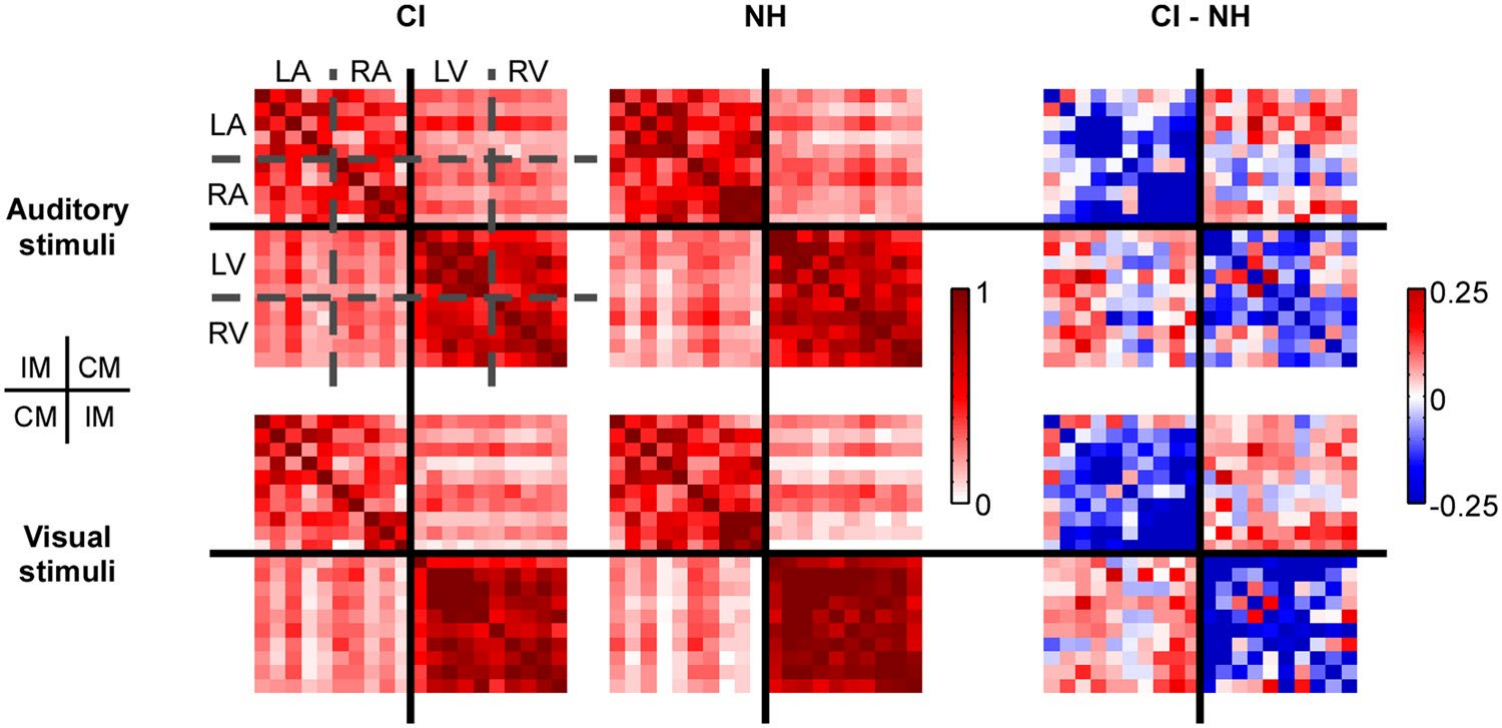
Functional connectivity correlation results. Correlation matrices are plotted separately for auditory (upper row) and visual stimuli (lower row), and separately for CI users (left column), NH controls (middle column) and the difference between CI users and NH controls (right column). Note the different scales used. Each correlation matrix has the layout as indicated in Fig. 1b, with the upper left and lower right quadrant containing intra-modal (IM) connectivity and the upper right and lower left quadrant containing cross-modal (CM) connectivity. The group difference indicates lower intra-modal connectivity and higher cross-modal connectivity in CI users compared to NH controls.

Figure 3 depicts the average correlation matrices obtained for CI users and NH controls in the visual and auditory stimulus condition. Consistently across both groups and both conditions, intra-modal connectivity (upper left and bottom right) was generally higher than cross-modal connectivity (upper right and bottom left). Additionally, the subtraction between CI users and NH controls demonstrated consistently lower intra-modal connectivity and consistently higher cross-modal connectivity in CI users for both visual and auditory stimuli.

The repeated measures three-way ANOVAs with factors stimulus type (auditory, visual), modality (cross-modal, intra-model) and group (CI, NH) for the right auditory area revealed a significant main effect of modality (F_1,36_ = 171.487, p < 0.0001, ŋ_p_^2^ = 0.826) with higher intra-modal connectivity than cross-modal connectivity, a significant main effect of stimulus type (F_1,36_ = 32.545, p < 0.0001, ŋ_p_^2^ = 0.475) with higher connectivity for visual than for auditory stimuli, a significant interaction between modality and group (F_1,36_ = 5.931, p = 0.020, ŋ_p_^2^ = 0.141), and a significant interaction between modality and stimulus type (F_1,36_ = 72.286, p < 0.0001, ŋ_p_^2^ = 0.668). The interaction between modality and group was the focus of the current study and thus was followed up by averaging between visual and auditory stimuli. The results showed significantly decreased intra-modal connectivity in CI users compared to NH controls (t_36_ = −2.227, p = 0.032), but no significant difference in cross-modal connectivity between the groups (t_36_ = −0.721, p = 0.475). Additionally, intra-modal connectivity was larger than cross-modal connectivity for both CI users (t_36_ = 9.761, p < 0.0001) and NH controls (t_36_ = 9.269, p < 0.0001). The result therefore shows that the interaction was driven by differences in intra-modal connectivity (Fig. 3).

**Figure 4:**
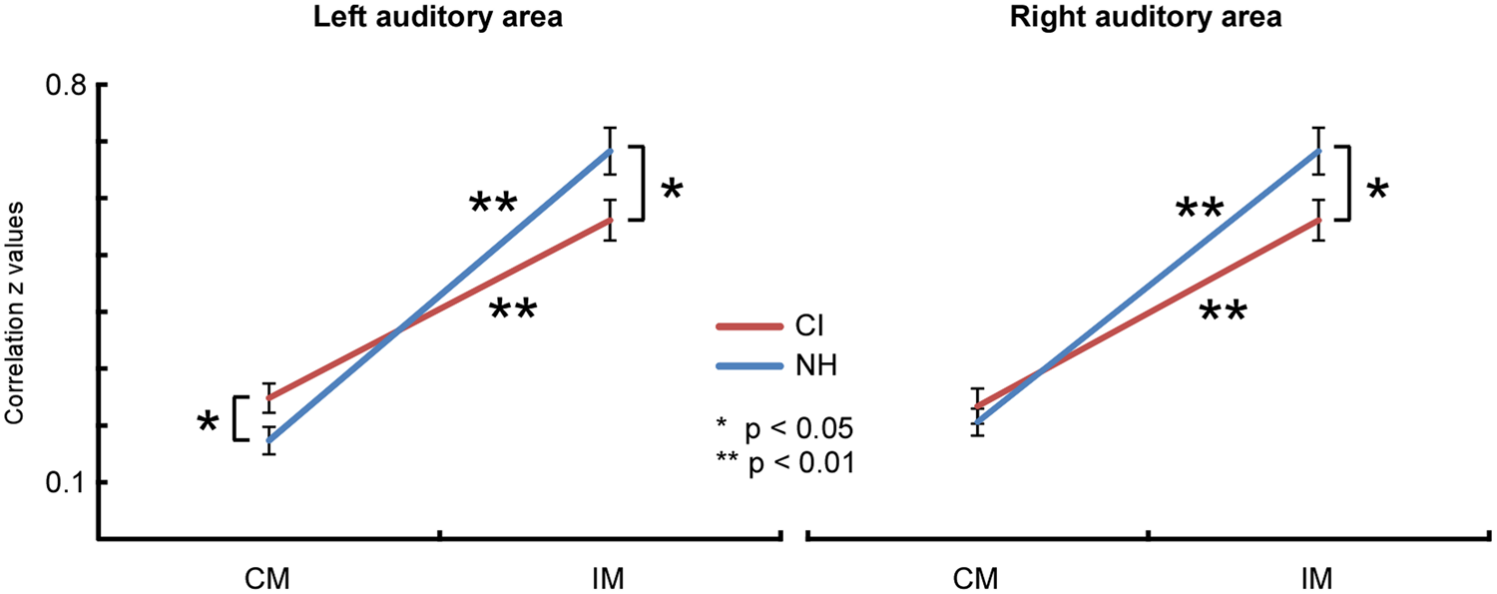
Group differences in intra-modal (IM) and cross-modal (CM) connectivity, averaged across stimulus type. Values for the left auditory area are shown on the left, and for the right auditory area on the right. Red lines represent CI users and blue lines the NH controls. The y-axis shows Fisher’s z-transformed correlations and the x-axis the modality. In the left auditory area, CI users had significantly decreased intra-modal connectivity and increased cross-modal connectivity.

The repeated measures three-way ANOVA for the left auditory area revealed a significant modality effect (F_1,36_ = 187.135, p < 0.0001, ŋ_p_^2^ = 0.839) with higher intra-modal connectivity than cross-modal connectivity, a significant stimulus type effect (F_1,36_ = 38.208, p < 0.0001, ŋ_p_^2^ = 0.515) with higher connectivity for visual than auditory stimuli, a significant interaction between modality and group (F_1,36_ = 10.660, p = 0.002, ŋ^2^ = 0.228), and a significant interaction between modality and stimulus type (F_1,36_ = 63.067, p < 0.0001, ŋ_p_^2^ = 0.637). Again the interaction between modality and group was followed-up with both stimuli averaged. The results showed significantly decreased intra-modal connectivity (t_36_ = −2.227, p = 0.032), as well as significantly increased cross-modal connectivity in CI users compared to NH controls (t_36_ = 2.103, p = 0.043, Fig. 4). Additionally, intra-modal connectivity was larger than cross-modal connectivity for both CI users (t_36_ = 10.035, p < 0.0001) and NH controls (t_36_ = 9.911, p < 0.0001).

To summarize, CI users compared to normal controls showed both a lower interhemispheric connectivity between left and right auditory areas in the auditory condition (Fig. 2b Corr_1) as well as a lower interhemispheric connectivity between left and right visual areas in the visual condition (Fig. 2b Corr_2). Additionally, for both visual and auditory conditions, the cross-modal functional connectivity between the left auditory area and both left and right visual areas (Fig. 2b Corr_3 and Corr_5) were found to be significantly higher in CI users compared to NH controls. No between-group differences were found between the right auditory area and both left and right visual areas (Fig. 2b Corr_4 and Corr_6).

**Figure 5:**
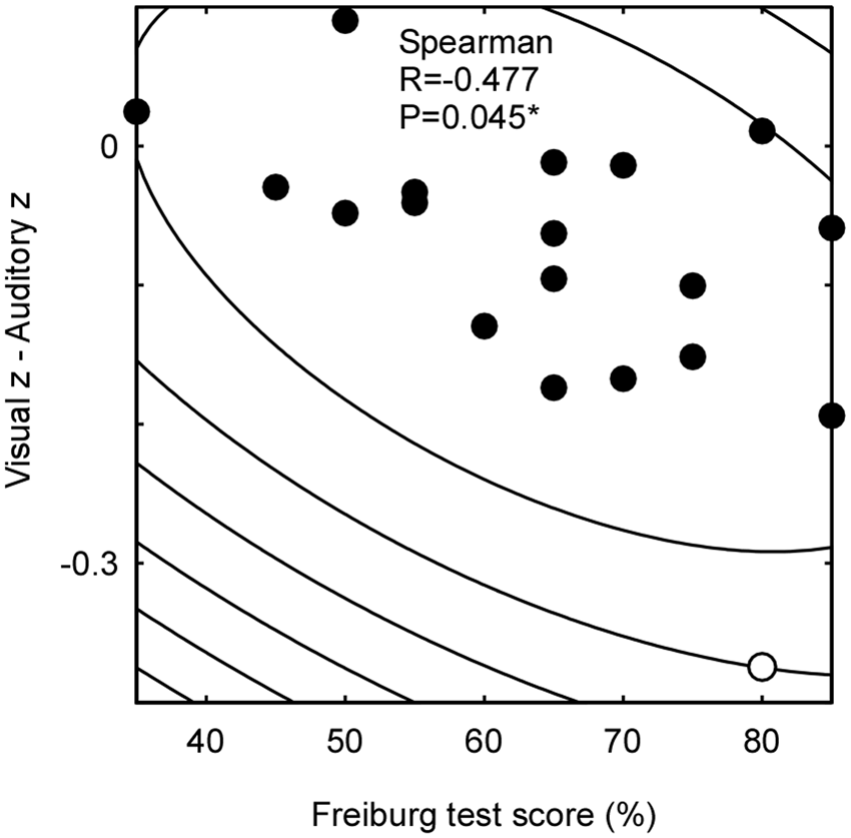
Correlation between speech performance and cross-modal connectivity. The y-axis shows differences between Fischer’s z-transformed correlation values for visual and auditory stimuli, reflecting cross-modal connectivity. The x-axis shows percentage correct scores from the Freiburg test, reflecting speech performance. The contour lines indicate the bootstrapped Mahalanobis distance *D*s from the bivariate mean. The hollow circle is an outlier whose average *D*s is 6 or greater. Higher speech recognition performance was associated with a greater difference between cross-modal connectivity for auditory and for visual stimulus.

To investigate the relationship between cross-modal connectivity and speech recognition ability, Fischer z-transformed correlation values for auditory stimuli were subtracted from those for visual stimuli and compared with speech recognition ability. A significant correlation was found showing that better Freiburg test scores in speech recognition were associated with more cross-modal connectivity for auditory stimuli than for visual stimuli (R = −0.525, p = 0.021). This association was robust against potential outliers. Outliers were identified via Mahalanobis distance and bootstrapping^44^. The correlation remained significant (R = −0.477, p = 0.045, Fig. 5) after removing the identified outlier. No correlation was found between the Fisher z-transformed correlation and the OLSA test scores. We also explored the correlation between functional connectivity and other parameters such as duration of deafness, duration of CI, and age. However, none of the correlations survived correction for multiple comparisons (alpha value at 0.01).

## Discussion

We compared intra-modal and cross-modal functional connectivity between a group of post-lingually deaf CI users and a group of age- matched NH controls. Post-lingually deaf CI users exhibited reduced intra-modal connectivity within visual and auditory areas on the one hand and greater cross-modal connectivity between visual and auditory areas in the left hemisphere on the other. In particular, the latter observation was found irrespective of the type of sensory processing (visual or auditory) involved. CI users thus recruited both auditory and visual cortices for processing both visual and auditory uni-sensory stimuli. Moreover, cross-modal functional connectivity was correlated with individual speech recognition skills in CI users. The current approach reveals important additional information which is complementary to the previous report. Specifically, the cross-modal activation in both visual and auditory cortices were not simply higher in CI users, they were also more synchronized temporally with the intra-modal activation. This additional temporal information strengthens the hypothesis that auditory cortex is recruited for visual processing and visual cortex is recruited for auditory processing in CI users.

Increased structural and functional connectivity has been previously observed in deaf animals and, likewise, in deaf humans^16, 24^. In particular, animal studies have revealed abnormally increased structural connectivity between visual and auditory sensory cortices not only in early deafness but also in adult-onset deafness^16, 17^. Thus the current finding of greater cross-modal functional connectivity in post-lingually deaf CI users may reflect left-over changes in connectivity previously induced during the period of deafness. However, previous animal studies focused exclusively on projections from visual to auditory cortex^15–17^ and little is known about possible projections from auditory to visual cortex. In contrast, our results indicate increased functional connectivity in CI users for processing both visual and auditory stimuli, which may suggest a bi-directional connection between visual and auditory cortices. Unfortunately, the fNIRS approach adopted here limits the observation to functional connectivity only, and the underlying anatomical pathways remain unclear. While the investigation of structural connectivity in CI users is not possible due to safety concerns, fNIRS provides a first opportunity to explore patterns of functional connectivity in CI users. Our current study was exploratory in nature and therefore does not allow us to draw strong conclusions. However, it provides first evidence suggesting altered cross-modal connectivity patterns in CI users, a finding that should be followed up in future studies. Future studies are encouraged to also explore the possibility of projections from auditory to visual cortex in both deaf and implanted animals. An auditory-to-visual connectivity study may shed light on whether sensory deprivation inflates bidirectional structural connectivity or whether hearing restoration with a CI results in systematic connectivity changes.

The visual and auditory tasks used in the present study were different in nature, since the visual task was an active discrimination task and the auditory task was a passive listening task while watching a silent video. However, one should note that the present study was not designed to compare between visual and auditory stimuli, but instead to compare between CI users and NH controls. Since both CI users and NH controls were presented with the identical stimuli, the imbalance between the visual and the auditory task was unlikely to influence the observed group difference. Another concern may be raised on the presence of visual stimuli in the auditory paradigm. While we cannot rule out a possible influence of visual stimuli in the auditory task on cross-modal connectivity, we argue that such influence would interfere with existing correlation since the auditory stimuli were temporally independent from the video stream. Supporting this argument, we found overall higher z values, which represents correlations, for visual than for auditory stimuli, suggesting that the presence of a video stream introduces potential interference to the correlation in the auditory session. Altogether, we believe that our observation of functional connectivity pattern changes in CI users compared to NH controls cannot be attributed solely to the presence of visual input during the auditory session. Nevertheless, more studies are required to confirm our findings.

There is clear evidence of cross-modal reorganization of both visual and auditory cortices in both deaf individuals and CI users^6, 9, 10, 12, 45^. However it remains unclear whether this reorganization is lateralized. Some studies have reported effects primarily in the right hemisphere^6, 10, 43, 45, 46^, others in the left^9, 47^, and some have reported no hemispheric differences at all^12, 48–50^. Several factors such as type of stimuli^47^ and experience with sign language^51^ have been suggested to contribute to this discrepancy. The current study identified increased cross-modal functional connectivity between the left auditory cortex and both right and left visual cortices. The result is consistent with a recent study showing increased functional connectivity between left auditory cortex and right visual cortex in the elderly with hearing loss^52^. Additionally, the effect in the left hemisphere may be related to the use of speech stimuli. Language processing is left-lateralized in most individuals^53–55^. However, whether or how these factors contribute to the cross-modal take-over remains to be investigated. More studies are required to systematically dissociate the potential influence of language processing on deprivation-induced and restoration-related functional connectivity changes in CI users.

On the other hand, a decreased intra-modal connectivity in CI users compared to NH controls was observed. For auditory processing, less temporal consistency was found between hemispheres in CI users. This result is consistent with the degraded auditory input with CIs and has also been previously reported in both animals and humans^17, 28^. However, decreased connectivity between hemispheres in visual areas for visual stimuli in CI users was also observed, which may seem rather surprising. Several other studies have also indicated changes in hearing-impaired individuals in visual processing. Specifically, mixed reports exist on whether visual activation in visual cortex may be lower or higher in deaf and CI users compared to NH controls^10, 21, 46, 48, 56, 57^. Furthermore, altered visual cortex activation may be related to improved visual abilities in deaf individuals. At least one study found an association between visual-evoked potential P1 amplitudes and faster visual detection response times in deaf individuals^58^. Additionally, another study found increased stimulus-specific adaptation for visual stimuli in CI users, which potentially suggests increased encoding efficiency for visual stimuli^38^. As a result, it may be that decreased functional connectivity between hemispheres in visual areas contributes to improved visual processing in CI users, such as during lip reading^11, 59, 60^. Unfortunately visual task performance was not assessed in our study, so it is not feasible to evaluate this hypothesis directly. Future study should follow up on this and determine whether decreased functional connectivity within visual areas serves as the underlying mechanisms for altered visual processing and performance in CI users.

Consistent with our hypothesis, cross-modal functional connectivity correlated with speech recognition. Specifically, individuals with higher cross-modal functional connectivity for auditory than for visual stimuli performed better than individuals exhibiting the opposite pattern. However, one should note that the current investigation revealed a correlation only with one out of three speech recognition tests performed. Unlike the OLSA tests measuring speech recognition in sentences, the Freiburg monosyllabic words test measures recognition of single words. Our result suggests that the observed changes in functional connectivity may be sensitive to only speech recognition in quiet without sentence structure information. Future studies are required to systematically investigate the relationship between functional connectivity patterns and different aspects of speech recognition. Additionally, the current correlation is consistent and complementary to our previous findings, suggesting that cross-modal activation in the visual cortex may be beneficial for speech recognition whereas cross-modal activation in the auditory cortex may be maladaptive. However, a few differences should be noted. Specifically in the previous report we used only auditory words in the correlation analysis, and in contrast both tones and words were used in the present study. Unfortunately since all the auditory conditions were randomly presented in sequence, the current experimental design did not allow us to investigate functional connectivity patterns for different type of auditory stimuli. Our results show on average increased cross-modal functional connectivity between visual cortices and the left auditory cortex, regardless of the type of auditory stimuli. Future studies should investigate systematically whether different type of auditory stimuli may have different influence on cross-modal functional connectivity.

Since communication in daily life often involves integration between visual and auditory modalities, the correlation result suggests that speech recognition based on degraded auditory input from a CI can be improved by recruiting visual areas. On the other hand, since visual perception of flickering checkerboards is not necessarily an inherently multisensory experience, it may not be beneficial to recruit auditory cortex for processing simple visual stimuli, hence the negative correlation between functional connectivity for visual stimuli and speech recognition^9, 10^. Note however that there may still be a functional purpose for increased cross-modal functional connectivity for visual processing. Our group recently reported a positive correlation between cross-modal activation in auditory cortex for face stimuli and lip reading^46^. Thus increased cross-modal connectivity for visual processing may facilitate lip reading and thereby support daily life communication in CI users. In other words, future studies should jointly consider both aural speech recognition and lip reading ability to better estimate CI benefit and the relationship with cross-modal reorganization in CI users^3^.

## Conclusions

The current study provides first evidence of altered cross-modal functional connectivity in CI users. Increased cross-modal functional connectivity between visual and auditory areas and decreased intra-modal connectivity between hemispheres was found when CI users were compared to NH controls. This finding extends the observation of cross-modal reorganization in both visual and auditory cortices, by indicating increased information flow between the sensory cortices. Structural connectivity cannot be assessed in CI users. The current functional connectivity study may have identified a mechanism underlying cross-modal reorganization in post-lingually deaf CI users. Changes in functional connectivity could serve as a new, complementary tool for predicting clinical outcome after cochlear implantation.
